# Development and implementation of a risk assessment tool for broiler farm biosecurity and a health intervention plan in the Netherlands, Greece, and Cyprus

**DOI:** 10.1016/j.psj.2022.102394

**Published:** 2022-12-09

**Authors:** Janneke Schreuder, Maro Simitopoulou, Kyriacos Angastiniotis, Paolo Ferrari, Maaike Wolthuis-Fillerup, George Kefalas, Sotiris Papasolomontos

**Affiliations:** ⁎ Animal Health & Welfare, Wageningen Livestock Research, WD Wageningen, 6708, The Netherlands; † VitaTrace Nutrition Ltd., Nicosia, 2033, Cyprus; ‡ Nuevo SA, Shimatari Viotias, 320 09, Greece; § CRPA Research Centre for Animal Production Reggio Emilia, 42121, Italy

**Keywords:** broiler, biosecurity, risk-based scoring tool, health plan

## Abstract

Preventing pathogens from entering and spreading on farms is the first step in reducing health problems. For this study a BiosEcurity Assessment Tool was developed to identify strengths and weaknesses in biosecurity on broiler farms, which was used as a starting point to formulate tailor-made health plans to improve broiler health and reduce antimicrobial use. Farms were divided into 3 separate areas according to associated biosecurity risk; high disease risk external areas (red zone), medium risk service areas (orange zone), and the clean and highly secure access-restricted green zone. In the Netherlands, Cyprus, and Greece, 13, 15, and 7 broiler houses were monitored for 4 production cycles (2 preintervention and 2 postintervention cycles). At the start of the study the BiosEcurity Assessment Tool assessment was performed and a health plan was made in consultation with the veterinarian. After the second cycle a start was made with the implementation of the health plan. Overall, the biosecurity level in the green and orange zones were significantly higher in the Netherlands compared to Greece and Cyprus, but there was no difference for the red zone or the transition zones between the countries. The interventions in the health plans were mostly directed towards those measures that could be implemented in the short term and with low costs in the green zone. In Cyprus a decrease in antimicrobial use was found postintervention. This was not the case in Greece and the Netherlands. In Cyprus and Greece footpad lesion improved after interventions were implemented, although this may have been an effect of season. In Dutch farms no improvement was detected, but both antimicrobial use and footpad lesions were lower at the start of the study compared to Cypriot and Greek farms. In conclusion, the BEAT shows to be a promising tool to assess biosecurity risks on broiler farms. The biosecurity assessment in combination with the farm specific health plans could contribute to antimicrobial reduction on broiler farms.

## INTRODUCTION

Preventing pathogens from entering and spreading on broiler farms is the first step in reducing health problems and improving animal welfare (Newell et al., 2011; Gelaude et al., 2014). Biosecurity measures are designed to prevent the introduction and spread of pathogens into a flock or herd. Routine biosecurity protocols should therefore be implemented in animal production systems. The adoption of these measures will not only significantly reduce the risk of disease introduction, but may also reduce the magnitude of the financial losses that may occur following infection in a flock (Nespeca et al., 1997).

Furthermore, improvement of biosecurity can contribute to a reduction in antimicrobial use (Chauvin et al., 2005; Stygar et al., 2020; Mallioris et al., 2022). In the Netherlands for example, reduction in antimicrobial use was achieved through the adoption of both stricter legislation and benchmarking and herd health and treatment plans (Speksnijder, 2017). Moreover, Mallioris et al. (2022) found that internal biosecurity as a whole had the largest impact on antimicrobial use in pig farms, highlighting the importance of good biosecurity practices for public health as well.

While biosecurity is perceived as an important part of on-farm management and is advocated as a norm for all animal based industries, commitment and full engagement in such protective or preventative action is not always the norm (Kristensen and Jakobsen, 2011; Racicot et al., 2011; Mankad, 2016). Effective biosecurity is closely linked to husbandry practices, and can be improved through the development and implementation of effective herd health and welfare plans. Although the health plans do exist (and are compulsory in some European Union member states) their use is often limited to an ineffective “tick box exercise” and compliance can sometimes be low (Nespeca et al., 1997; Mankad, 2016). For example, a report by the Australian government cited a gap in current biosecurity engagement in terms of adequate monitoring and evaluation of threat, risk and on-farm practices (Kruger et al., 2009). To strengthen biosecurity on farms, identification is needed of farm-specific success and risk factors for biosecurity. When risk factors are identified, tailor-made health plans for each specific farm can be made and this will make it easier to also monitor improvements and generate “alerts” when necessary (Caekebeke et al., 2020).

This study describes the development of a novel biosecurity protocol on the basis of the FAO 3-Zone biosecurity model (FAO, 2015) combined with information from biocheck.ugent (Laanen et al., 2010; Gelaude et al., 2014), and Dutch Hygiene scoring system for poultry farms (AVINED, 2022). This biosecurity assessment tool (**BEAT**) was used to identify farm specific strengths and weaknesses with regards to biosecurity for each zone by the farmer and their allied veterinarian. Based on these insights, the farmer and veterinarian can make a tailor-made farm-specific health plan for each zone to strengthen on-farm biosecurity. Involving the veterinarian in the biosecurity assessment and draw-up of health plans was suggested to improve compliancy to biosecurity (Mankad et al. 2016) and is what distinguishes the BEAT from other biosecurity tools. The BEAT was then pilot tested on farms in the Netherlands, Greece, and Cyprus and technical performance parameters, as well as antimicrobial use and footpad lesions were monitored to assess if the implementation of the health plans resulted in improved health and a reduction of antimicrobial use.

## MATERIALS AND METHODS

### Poultry Farms

Seven, 15, and 13 broiler houses were recruited to participate in this study in Cyprus, Greece and the Netherlands respectively. Farms were selected for the study based on recent (previous 5 production cycles) use of antimicrobials on their farms, and farmers had to be willing to participate in this research and perform the BEAT assessment with the farm veterinarian. As such, the sample of farms participating was not representative of the broiler farms in each country. Due to difficulties recruiting farms in the Netherlands with antimicrobial use in their recent history, some farms (n = 3) with no antimicrobial use had to be involved. Each house was located on a different farm, except in Greece where some houses were on the same farm (n = 15 broiler houses and n = 10 farms; Supplemental Table S1). The broiler houses on the same farm had different management and antimicrobial history and thus were considered as independent. In the Netherlands, all farms worked with their own farm veterinarian, which was the same for all production cycles. All Greek farms worked with the same farm veterinarian and the same was true for the Cypriot farms. As all farms were on different locations, these were considered as independent farms. All farms had Ross 308 chickens and were monitored between December 2019 and March 2021 (Supplemental Table S1).

### BEAT Assessment and Health Plans

A risk assessment tool (BEAT) was developed, based on the 3-Zone Biosecurity model from the FAO (FAO, 2015), a risk-based scoring system developed by Ghent University, the Biocheck.ugent (Gelaude et al., 2014) and the Dutch Hygiene scoring system for poultry farms, that is, “Hygiene scan” (AVINED, 2022) and complemented with literature review (Supplemental data S2). This was a scan of most common pathogens in broilers and risk factors associated with introduction on broiler farms. The format of the BEAT anticipated on the format of the health plans to be worked out, which followed the zoning systematic (red, orange, and green). The zones were defined according to the associated biosecurity risk; high disease risk external areas (red zone; all external areas), medium risk service areas (orange zone; paved farmyard and functional areas), the clean and highly secure access-restricted green zone containing the actual broiler house where the chicken flock is located. Green zones on poultry farms contain the broiler house and entry room where present; the orange zone, also called the professional zone, is the usually paved area of the farm in between the farm gate and the broiler houses, where staff, vehicles, and machineries circulate; the red zone contains the external areas (unpaved roads, ditches, pastures etc.). For each zone, questions were formulated which were based on most important risk factors for disease introduction and associated preventive measures (Supplemental data S2). For each question, predefined answer possibilities were given, which were linked to a point distribution system ranging from 0 to 1 (fully compliant 1 up to no compliance 0). The point distribution corresponded with green (1, high score) to red (0, low score), to make it easily identifiable where the strengths and weaknesses were for the farmer and the veterinarian. The points per zone were summed which resulted in a total and relative BEAT score per zone for each farm. The BEAT was made in excel (Supplemental data S3).

**Figure 1:**
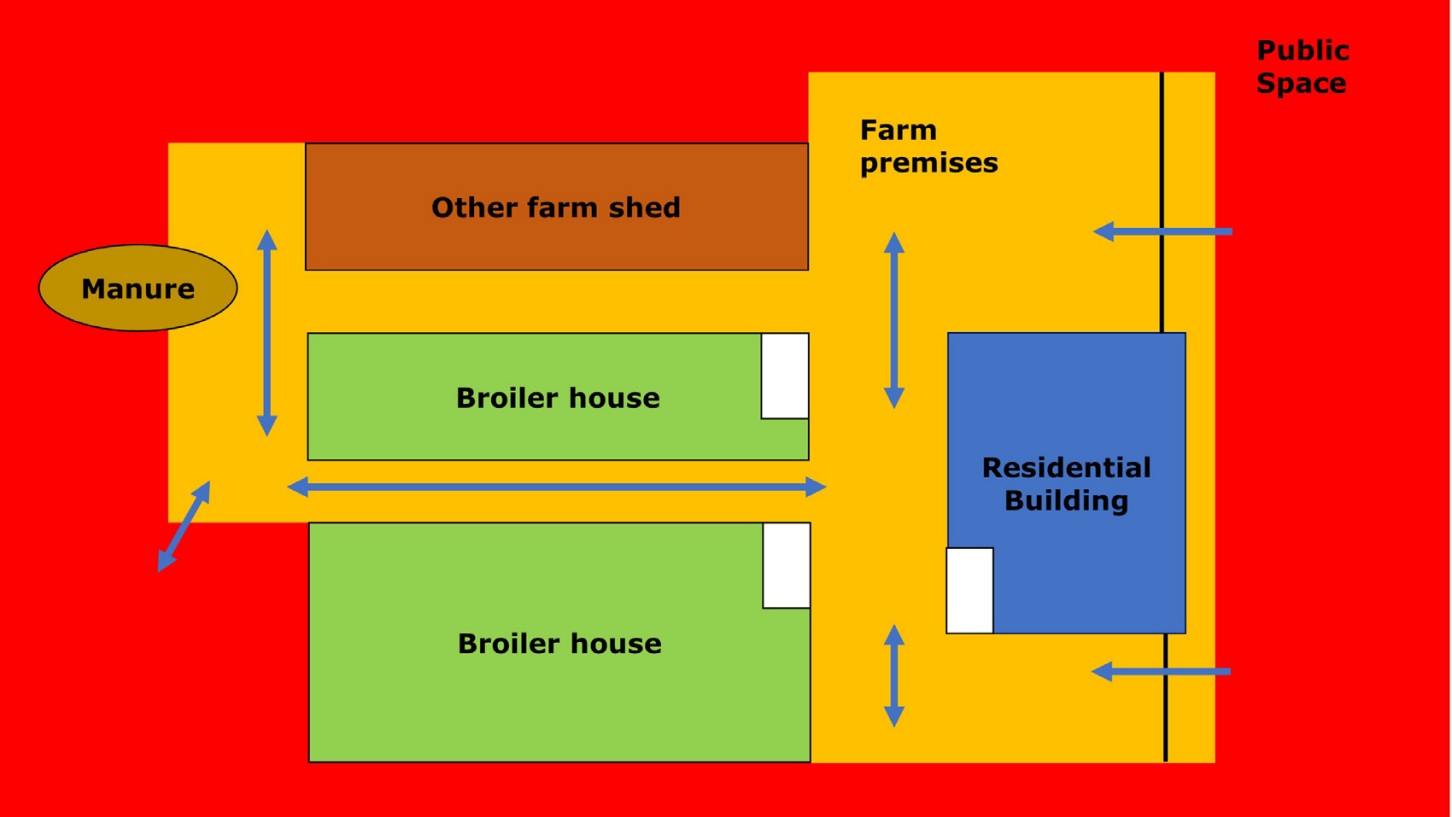
Overview of zoning systematic on poultry farms. Red, orange, and green zones are identified. Arrows indicate main traffic routes.

In short, broiler houses were followed-up for 4 production cycles (cycles 1–4), of which 2 cycles were considered as preintervention and 2 cycles as postintervention. At the start of cycle 1 the BEAT was carried out by the farmer and their veterinarian. As a first step the farmers and their veterinarians first identified and colored the different zones using a Google Earth image of the farm property, after which a schematic drawing of the farm location was made with the risk zones, the main buildings, stables, storage sites, pathways etc. (Figure 1, Supplemental data S3). The second step was to evaluate the biosecurity of each zone and its transition zone systematically through the list of questions in the BEAT tool, which resulted in a relative beat score per zone. With the output of the BEAT assessment, a health plan was made for each farm by the farmer and veterinarian, which is described below. These health plans were implemented after the second cycle and evaluated after the fourth cycle.

### Health Plans

The health plan contained a list of interventions per zone which were aimed at strengthening on-farm biosecurity, and were formulated in a SMART way (Specific, Measurable, Achievable, Relevant, and Time-bound). The draft health plan was then checked by the researcher and returned to the farmer and veterinarian with feedback, so that the farmer could start with the implementation of the interventions after the second cycle. For processing of the data, the farmer and veterinarian estimated if the interventions could be implemented on the short, medium or long term as well as if costs for implementation of the interventions were low, medium or high. At the end of the fourth cycle, farmer and veterinarian determined whether interventions had been implemented or not (realized or not realized) and the researchers assigned each intervention in the health plans to a category of a zone of the BEAT tool.

### Sample and Data Collection

Besides the risk assessment the following samples were collected for each house for each cycle during the period of the study: details of any antimicrobial use and days of treatment were recorded, as well as technical production parameters concerning mortality, slaughter weight, slaughter age, and more (Supplemental Table S1). Furthermore, after each cycle footpad lesions were scored as described by Berg (1998), as it is considered an important welfare indicator in broilers (Meluzzi et al., 2008).

### Statistical Analyses

All analyses were performed in R 4.1.0 (R Core Team, 2021). For this study, the relative BEAT scores are given per zone across all countries, per zone per country and per country per category (Table 1). In the manuscript, average relative BEAT scores are shown with standard deviations (±SD). First, overall differences in relative BEAT scores between zones across all farms are shown. To assess differences between countries in relative BEAT scores per zone, Wilcoxon-Rank-Sum tests were performed. To analyze if there were differences in mortality and footpad lesions pre- and postintervention these were first analyzed using a Kruskal-Wallis test. To further disentangle effects, footpad lesion were analyzed in a linear mixed effects model, with country as random effect and cycle number, the month in which the cycle started (to check for seasonality which could be of influence on the footpad lesion scores) and construction year (older buildings may not have the best climate and environment, which could be of influence on the footpad lesion scoring) as explanatory variables using the lme4 package (Bates et al., 2015). Model selection was performed with forward selection based on Akaike's Information Criterion, with the lowest Akaike's Information Criterion indicating the best fit (Blanchet et al., 2008), and the final model contained country as random effect and the month in which the cycle started as fixed effect. To determine antimicrobial use per cycle, it was first determined whether flocks were treated with antimicrobials in a cycle or not. For the flocks with antimicrobial use, the average number of treatment days of the flocks was calculated per country and per pre- or postintervention cycle. Due to the limited number of farms using antimicrobials per country, we only show descriptive statistics for this part.

**Table 1:** Overview of number of interventions in the health plans of farms attributed per category as identified by the BEAT assessment and shown per zone and country.

		The Netherlands			Cyprus			Greece			
Risk category	Zone	Interventions	Realized	BEAT score	Interventions	Realized	BEAT score	Interventions	Realized	BEAT score	Total BEAT
Introduction of purchased animals	Green	0		0.65	7	7	0.66	15	0	0.13	0.43
Introduction by bedding/enrichment materials	Green	0		1.00	0		1.00	0		1.00	1.00
Introduction by contaminated feed	Green	0		0.98	7	0	0.00	15	0	0.50	0.59
Introduction by drinking water	Green	1	1	0.76	21	19	0.68	60	30	0.63	0.69
Thinning	Green	2	1	0.14	14	0	0.05	45	0	0.00	0.07
Depopulation	Green	0		0.18	0		0.08	0		0.00	0.09
Spread of pathogens between consecutive flocks	Green	2	2	0.85	7	2	0.82	15	0	0.58	0.73
Spread between farm broiler houses	Green	3	3	0.57	7	0	0.67	15	0	0.87	0.71
Removal of dead birds from the house	Green	5	3	0.64	7	1	0.50	0		1.00	0.76
Rodents/insects	Green	2	1	0.93	0		0.89	0		1.00	0.95
Wild birds	Green	0		0.93	7	2	0.29	0		0.20	0.50
Outdoor broiler areas	Green	0		0.42	0		1.00	0		0.60	0.61
Cleaning and disinfection of entree room	Green	0		0.80	0		0.35	15	15	1.00	0.80
Cleaning and disinfection of broiler house	Green	1	1	0.95	0		1.00	30	0	1.00	0.98
Total Green	Green	16	12		77	31		210	45		
Access of personnel/visitors	Orange-green	22	19	0.65	35	10	0.21	60	45	0.75	0.61
Access of materials	Orange-green	0		0.51	0		0.50	0		0.12	0.34
Access of wild birds (and pest animals)	Orange-green	0		0.64	0		0.72	0		0.62	0.65
Total orange-green		22	19		35	10		60	45		
Position of broiler houses relative to internal-external logistic lines	Orange	2	2	0.64	7	0	1.00	15	0	0.67	0.72
Cadaver storage	Orange	0		0.96	0		0.50	0		0.50	0.68
Manure storage	Orange	0		0.86	0		1.00	0		0.53	0.75
Feed storage	Orange	2	2	0.75	7	0	0.50	15	0	0.50	0.60
Storage of bedding materials	Orange	1	0	0.79	0		1.00	15	0	0.00	0,50
Other poultry species	Orange	0		0.89	0		1.00	0		0.75	0.85
Other farm animal species	Orange	0		0.63	0		1.00	0		0.72	0.74
Rodents/insects	Orange	0		0.85	0		0.67	15	0	0.33	0.60
Wild birds	Orange	0		0.79	0		0.72	15	0	0.30	0.57
Contaminated farm yard surfaces	Orange	0		0.96	0		0.71	0		0.00	0.51
Cleaning and disinfection of farm yard	Orange	2	2	0.68	7	0	0.22	1	0	0.49	0.51
Total Orange		7	6		21	0		76	0		
Access of personnel/visitors	Red-orange	11	7	0.53	7	2	0.53	6	0	0.74	0.62
Access of transport vehicles	Red-orange	4	4	0.38	7	2	0.64	15	0	0.02	0.28
Access of wild birds (and pest animals)	Red-orange	2	1	0.74	0		0.67	0		0.64	0.69
Separation orange and red zone by fence/wire and entrance gate	Red-orange	3	2	0.79	7	4	0.57	3	0	0.07	0.44
Arrival sign	Red-orange	4	2	0.57	0		0.71	0		1.00	0.78
Registration of visitors	Red-orange	1	1	0.86	7	5	0.00	0		1.00	0.75
Total Red-Orange		25	17		28	13		24	0		
Poultry density in area	Red	0		0.79	0		0.29	0		0.13	0.42
Distance to nearest poultry farm	Red	0		0.64	0		0.36	2	0	0.43	0.50
Shortest distance to public road with daily animal transports	Red	0		0.30	0		0.57	0		0.13	0.28
Spread of poultry litter/manure on surrounding fields	Red	0		0.80	0		1.00	1	0	0.56	0.74
Spread of other farm animal litter/manure on surrounding fields	Red	0		0.26	0		0.04	0		0.71	0.40
Mowing of premises	Red	1	1	0.23	0		0.21	0		0.50	0.34
Ploughing in surrounding fields	Red	0		0.45	0		0.07	0		0.50	0.40
Water ponds present within radius of 1 km	Red	0		0.43	0		0.57	0		0.87	0.64
Migratory birds route in the vicinity within radius of 1 km	Red	0		0.71	0		0.71	0		0.93	0.81
Pest animal pressure in surroundings	Red	0		0.75	0		0.21	0		0.00	0.33
Parking areas visitors/farm employees in red zone	Red	4	3	0.43	0		1.00	0		1.00	0.78
Separation "dirty"- "clean" area: location of dirty road in red zone	Red	2	1	0.29	7	7	0.48	15	0	0.33	0.34
Total red		7	5		7	7		18	0		0.43

The number of realized interventions (Realized) per risk category and the average relative BEAT score (BEAT score) per category of the farms are shown, and the average relative BEAT score per country for each risk category.

The total BEAT is the average relative BEAT score of that risk category across all countries. Per zone, the total number of interventions is also indicated.

Abbreviation: BEAT, BiosEcurity Assessment Tool.

## RESULTS

### BEAT Scores

**Figure 2:**
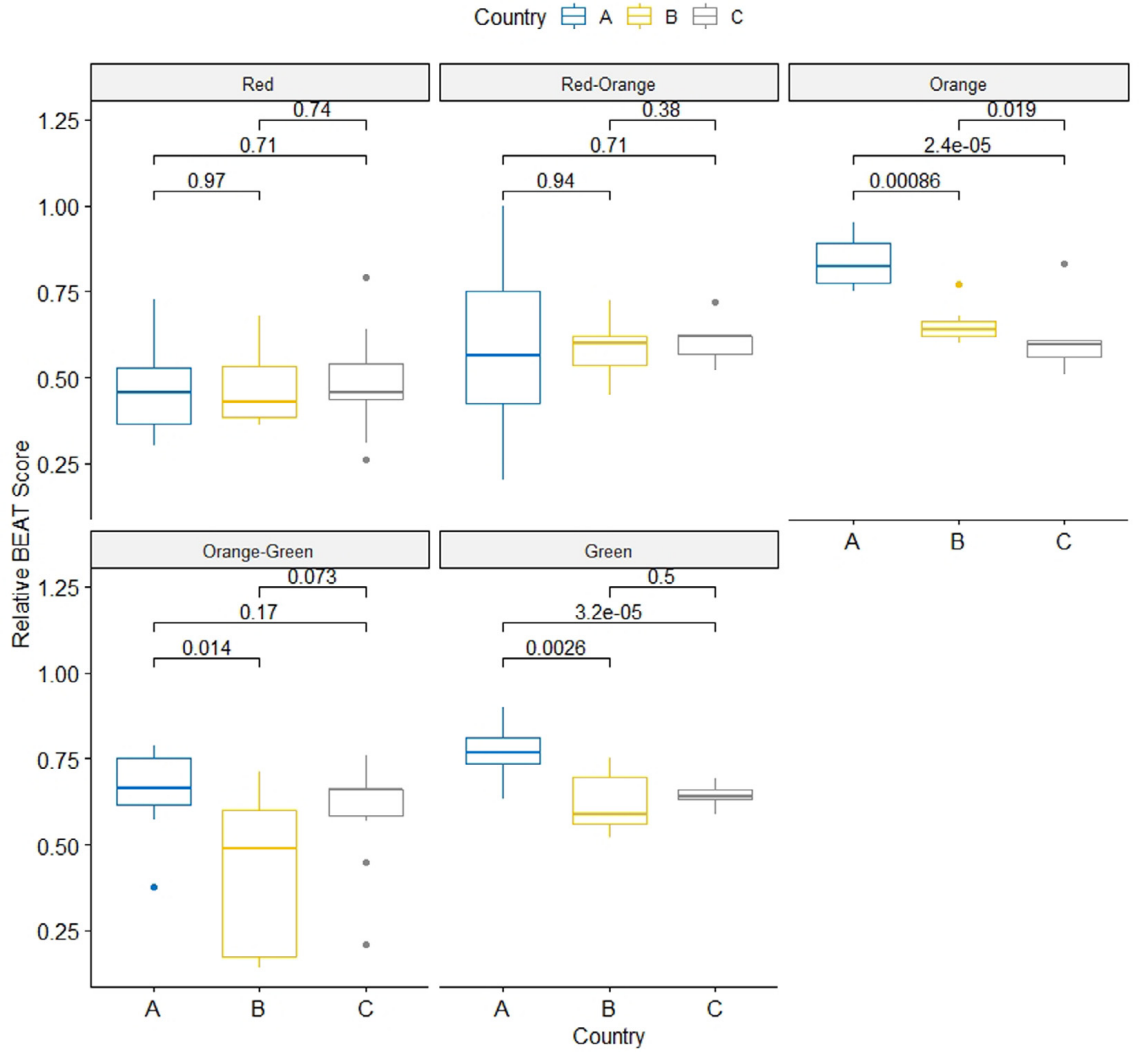
Average relative BEAT-scores per zone, shown per country (A) the Netherlands, (B) Cyprus, (C) Greece. *P*-values (Wilcoxon-Rank-Sum test) are shown to indicate differences between countries. Abbreviation: BEAT, BiosEcurity Assessment Tool.

Across all countries, the BEAT score in the red zone was lower compared to the other zones (Supplemental Figure S1). Higher (and thus better) BEAT scores of the orange, orange-green, and green zone were found in farms from the Netherlands compared to farms from Cyprus (*P* < 0.01 green zone; *P* < 0.05 orange-green zone; and *P* < 0.01 orange zone) and Greece (*P* < 0.01 green zone; *P* < 0.01 orange zone). There was no difference within different zones between Cyprus and Greece (Figure 2).

In the green zone, scores were lowest in categories “Thinning” (0.07 ± 0.08 average relative BEAT-score) and “Depopulation” (0.09 ± 0.09 average relative BEAT-score; Table 1). In the orange-green zone the lowest scores were assigned to “Access of materials” (0.34 ± 0.37 average relative BEAT-score) and in the orange zone the category with the lowest score was “Storage of bedding material” (0.50 ± 0.51 average relative BEAT-score). In the red-orange transition zone “Access of transport vehicles” and “Separation orange and red zone by fence/wire and entrance gate” had the lowest scores (0.28 ± 0.35 and 0.44 ± 0.50 average relative BEAT-scores respectively). In the red zone “Shortest distance to road” (0.28 ± 0.37 average relative BEAT score), “Mowing of premises” (0.34 ± 0.37 average relative BEAT-score), “Separation 'dirty'- 'clean' area: location of dirty road in red zone” (0.34 ± 0.17 average relative BEAT-score) and “Pest animal pressure” (0.33 ± 0.43 average relative BEAT score) all scored below 0.4 (Table 1).

### Health Plans and Interventions

The number of interventions that were assigned to each risk category per zone of the BEAT are shown per country and across all countries in Table 1. The average BEAT score for that category for each country and the number of interventions that were realized at the end of the study after cycle 4 are also shown. Most interventions were planned for the green zone (303), followed by the orange-green zone (117), the orange zone (104), the red-orange transition zone (77), and the red zone (32; Table 1). Of the interventions, 75.2, 36.3, and 23.0% were realized after cycle 4 in the Netherlands, Cyprus and Greece respectively. Of all interventions, most were interventions that could be realized on the short term and were low in cost (n = 222; Table 2). The realization rate at the end of the study was highest for low cost – short/medium term interventions (53 and 51% respectively), and lowest for interventions with high costs and long term (1%; Table 2).

**Table 2:** Number of interventions as shown per cost estimate and amount of time it takes to realize.

Term\costs	Low	Medium	High
Short	222 (53%)	44 (43%)	0
Medium	88 (51%)	94 (12%)	33 (21%)
Long	0	44 (20%)	111 (1%)

In brackets the percentage of interventions that was realized at the end of the study period is shown.

### Biological Data and Antimicrobial Use

**Figure 3:**
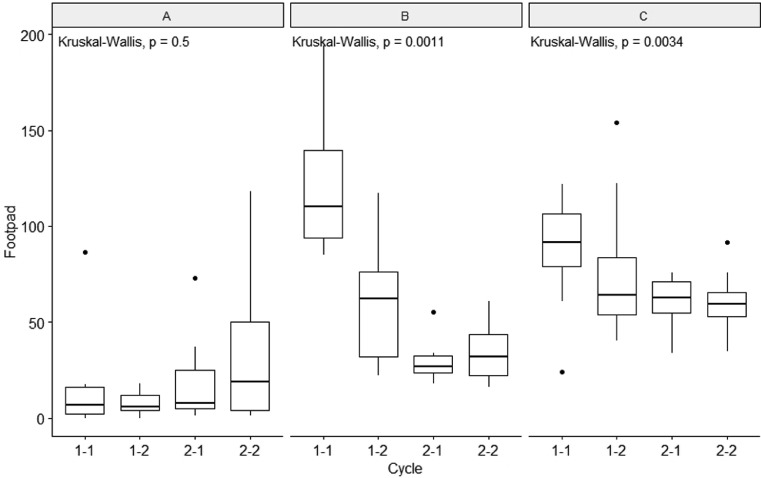
Footpad lesion scores shown per country (A) the Netherlands, (B) Cyprus, (C) Greece for the different cycles that were followed up. Cycles 1-1 and 1-2 were preinterventions cycles; Cycles 2-1 and 2-2 were postinterventions cycles.

Lower footpad lesion scores were found in postintervention cycle numbers in Greek and Cypriot farms (*P* < 0.01, Kruskal-Wallis, Figure 3). However, no significant differences between cycle numbers were found in the final model corrected for seasonality (Figure 3). Footpad lesions overall were lower in the Netherlands, compared to Greece and Cyprus (*P* < 0.01). There was no difference in broiler mortality across cycle numbers in any of the countries (*P* > 0.05, Kruskal Wallis). On average, a lower number of flocks was treated with antimicrobials in Dutch farms (42%) compared to Cyprus (60%) and Greece (100%) (Table 3). In 3 out of 13 farms in the Netherlands, no antimicrobials were used throughout the study, while all farms from Greece (n = 15) and Cyprus (n = 7) used antimicrobials in at least 1 cycle. In farms from Cyprus, more farms (n = 4 and n = 5 postintervention cycles 1 and 2 respectively) did not use any antimicrobials after the second cycle as compared to preintervention (n = 1 for both preintervention cycles), whereas this was not found in Dutch and Greek farms (Table 3). The mean days of treatment when antimicrobials were used in a cycle did not decrease in postintervention cycles compared to preintervention cycles in any of the countries (Table 3).

**Table 3:** Antimicrobial use (AMU) described as the number (#) of farms with AMU in each cycle per country and the median (mean) days of treatment of farms with AMU per cycle.

Country	AMU	Cycle 1	Cycle 2	Cycle 3	Cycle 4
The Netherlands	# farms with AMU	3	7	8	4
	Median days of treatment (mean)	5 (5.3)	3 (3.8)	3 (3.5)	4 (5.6)
Cyprus	# farms with AMU	6	6	3	2
	Median days of treatment (mean)	11 (10.7)	4.5 (5.8)	7 (6.0)	6 (6.0)
Greece	# farms with AMU	15	15	15	15
	Median days of treatment (mean)	7 (6.4)	5 (4.6)	6 (6.0)	6 (5.9)

For reference, a total number of 13 farms in the Netherlands, 7 farms in Cyprus, and 15 farms in Greece were included in the study.

## DISCUSSION

This study describes the use of a novel BEAT, the BEAT, to identify strengths and weaknesses in biosecurity on broiler farms based on the FAO 3-zone model. This assessment was used as a base to formulate tailor-made health plans for farms in the Netherlands, Greece, and Cyprus aiming at improving animal health and as a consequence, reduce antimicrobial use.

Across all countries BEAT scores were lowest for the red zone. Furthermore, there was a difference in BEAT scores of the green and orange zones between farms from the different countries, for which Dutch farms had higher BEAT scores compared to Greek and Cypriot farms. This difference might be explained by the fact that broiler farmers from Dutch farms already apply a yearly hygiene scan (AVINED, 2022) as part of a quality assurance system for the Dutch poultry industry. Since 2014, this hygiene scan is filled in once yearly by the farmer, the veterinarian however is not involved. Furthermore, in the Netherlands recent bird flu outbreaks have likely made farmers more aware of the importance of biosecurity, as biosecurity plays a key role in preventing avian influenza outbreaks (Ssematimba et al., 2013), and as a result might explain why BEAT scores were higher. This phenomenon was reported previously in poultry flocks in Georgia (United States) in which farms that recently had an outbreak of infectious laryngotracheitis had higher biosecurity levels (Dorea et al., 2010). In contrast, there was no difference in BEAT scores of the red zone among the 3 countries. The red zone mostly comprises external factors, which are not easily influenced by the farmer, except for deciding the farm location in case of a new farm.

Within the green zone, categories depopulation and thinning scored the lowest across all countries. Thinning is a common practice in many European countries and is considered a risk for pathogen transfer, like *Campylobacter*, due to breach of biosecurity measures as this involves animal transport vehicles and also a catching team to enter the farm premises (Hege et al., 2002; Smith et al., 2016; Hertogs et al., 2021). This result is in line with another study on biosecurity levels in conventional broiler houses in Europe, where subcategory depopulation also scored relatively low (Van Limbergen et al., 2018). Although thinning practices were not stopped on the farms, many interventions were aimed at informing the catching crew around thinning about proper hygiene protocols, which does contribute to reduce the risk of pathogen introduction during thinning (Hertogs et al., 2021). Overall, most interventions were aimed at reducing risks in the green zone. This is to be expected, as the green zone entailed most behavioral aspects that are easily implemented and realized.

For the orange and orange-green transition zones, access of materials and storage of bedding materials scored lowest across all countries. When materials (either equipment or bedding) are not properly protected or disinfected before entering the broiler house, this can be a risk to transmit pathogens (like avian influenza) into the broiler house (Conraths et al., 2016). Many interventions were aimed at reducing this risk, like proper disinfection of materials and making sure rodents and wild birds are unable to reach the bedding material. In line with this, many interventions were also aimed at improving feed storage and making sure rodents and wild birds cannot reach them to prevent contamination of the feed, as well as disinfection of the farm properties, to prevent that pathogens from a contaminated farm yard can enter the poultry farms via vectors (e.g., rodents) or fomites (Meerburg and Kijlstra, 2007; Shriner et al., 2016; Velkers et al., 2017).

Overall the lowest BEAT scores were reported for the red zone, where shortest distance to public road with animal transport, mowing of premises, pest animal pressure in surroundings, and separation of dirty-clean area all scored < 0.4. Moreover, the least interventions were aimed at the red zone and the interventions that were aimed at the red zone were mostly aimed at improving the separation of the dirty and clean area. The red zone mostly comprises external factors, which are not easily influenced by the farmer.

A decrease in antimicrobial use in farms from Cyprus was seen postintervention, where 4 (postintervention cycle 1) and 5 (postintervention cycle 2) out of 7 farms did not use antimicrobials during a cycle compared to 1 (for both pre intervention cycles) out of 7 preintervention. Given the short study period and the low number of farms, this result looks promising, although reduction might have been a result of the so called Hawthorne effect which concerns research participation and the consequent awareness of being studied, and the possible impact on behavior as a result (McCambridge et al., 2014). In this case, participation to the study might have made the Cypriot farmers and veterinarian more aware of antimicrobial use, which affected antimicrobial use and prescription behavior. Dutch and Greek farms did not show a change in antimicrobial use over time. In the Netherlands, antimicrobial use was already low at the start of the study. Between 2009 and 2019 antimicrobial use by Dutch farms (broilers, pigs, veal calves, and dairy cattle) has been reduced by approximately 69.6% (Mallioris et al., 2022), which explains why antimicrobial use in the Dutch farms was lower to start with and might also explain why it was more difficult to detect a difference over time as a result of the study. Furthermore, in 2020, in only 23.0% of conventional broiler flocks in the Netherlands antimicrobials were prescribed (Royal GD, 2022), which indicates that it is fairly common for Dutch broiler farms to not use antimicrobials in a flock. In Greek farms, only 23.0% of the planned interventions on the health plans had been realized at the end of the study, which might explain why no effect was found on antimicrobial use. The lower compliancy on Greek farms might be explained by the fact that more interventions on Greek farms needed high cost investments compared to the interventions planned on Dutch and Cypriot farms. Only 1% of estimated high cost interventions were realized across the entire study, likely because these take a longer time to realize, which was not feasible within the period of this study.

Compliance to the health plans was higher in farms from the Netherlands (75.2%) compared to farms from Cyprus (36.3%) and Greece (23.0%). In the Netherlands more short term interventions with low costs, that is, measures that were more easily implemented, were formulated compared to Greece and Cyprus. Especially the interventions aimed at the long term and high costs had not been realized at the end of the study. Short term and low cost interventions mostly aimed at improving disinfection and training of people entering the farm. Research has shown that still a lot of biosecurity errors are made with people entering the farm (Racicot et al., 2011), while improving these errors are considered probably one of the easiest and least expensive ways to improve biosecurity on a broiler farm of which the costs are fairly low (Siekkinen et al., 2012; Niemi et al., 2016).

Footpad lesion scoring improved during the study period, however the influence of seasonality could not be ruled out. Studies show that seasonality, which can influence the moist in the litter, is a risk factor for footpad lesions (Ekstrand and Carpenter, 1998; de Jong et al., 2012; Kyvsgaard et al., 2013), with peak flock footpad lesion scores occurring in winter, whereas flocks placed in warmer summer months displayed lower flock footpad lesion scores.

The BEAT that was developed in this study, distinguished itself from other biosecurity tools in that it is based on the FAO zoning system. Where other biosecurity tools identify internal and external biosecurity (biocheck.ugent; (Gelaude et al., 2014)), the BEAT identifies 3 zones (red, orange, and green) and 2 transition zones and works with a map of the farm premises to identify each zone. This makes it easier to identify weaknesses per zone that could be improved and also clarifies where interventions should be placed. By involving the veterinarian, the exercise is not reduced to “ticking boxes,” thus improving compliance with the implementation of planned interventions. Previous research suggested that, to increase biosecurity compliance by famers, it is important that there is 1) evidence or at least perception that the recommended biosecurity responses are useful and effective, 2) the recommendations/call to action must come from a trusted and credible source and 3) a perception exists that other in the referent peer group are behaving in the same way (Hu et al., 2006; Mankad, 2016). By including the veterinarian in the assessment of the biosecurity status and making of intervention plans, the first 2 points are taken into account as the veterinarian is generally considered a reliable source to advice the farmer with the latest knowledge. For future research a longer follow-up period would be recommended to determine the compliancy to the health plans and measure the long term effect on antimicrobial reduction.

To summarize, this study shows that the novel BEAT assessment was a useful way to identify strengths and weaknesses regarding biosecurity on broiler farms and that the assessment with the tool is a good starting point for the making of tailor-made health plans for each farm. Furthermore, even considering the short follow-up period, we already report a reduction in antimicrobial use in one of the countries. A long term follow-up is needed to truly measure the effects. The results of this study are representative for the recruited farms and should be extrapolated with care, but will certainly be useful for implementation of additional practical measures to improve the biosecurity and health status in the broiler industry.

## Appendix Supplementary materials

Image, application 1

Image, application 2

Image, application 3

Image, image 4
