# Supplementary material for: Development and implementation of a risk assessment tool for broiler farm biosecurity and a health intervention plan in the Netherlands, Greece, and Cyprus
Source: Poult Sci. 2022 Dec 9;102(2):102394. doi: 10.1016/j.psj.2022.102394 (PMC9811215; doi:10.1016/j.psj.2022.102394)
Supplement: Supplementary file 1 [file mmc1.pdf]

Healthy Livestock, WP1
Internal Working Document: T1.1

Biosecurity en Biomarkers for broilers

Results of a quick scan on scientific and ‘grey’ literature

Final version 15-4-2019

Martien Bokma/Fleur Hoorweg/Jorine Rommers,

Wageningen Livestock Research, the Netherlands

Content

1. Introduction 3
2. Major infectious broiler diseases in the Netherlands 3
3. Literature scan: major diseases, transmission routes and prevention 4
3.1 Introduction 4
3.2 Overview of diseases, risk factors and potential preventative measures 4
3.3 General literature on biosecurity of broiler farms 5
3.4 Matrix of diseases and relevant subcategories of biosecurity 6
4. Existing scoring systems for biosecurity 7
4.1 Dutch scoring systems 7
4.2 Belgian scoring system 8
5. Risk zoning of broiler farms 9
6. Biomarkers 9
6.1 Gross list of potential biomarkers 10
6.1.2 Specific biomarkers: Digestive disorders 11
6.1.3 Specific biomarkers: Respiratory diseases 12
6.1.4 Specific biomarkers: Feet disorders 13
7. Follow-up 13
Literature 14

1. Introduction

WP1 of the Healthy Livestock Program will quantify the main housing and management risk factors associated with disease entering and spreading on, among others, broiler farms, and will define biomarkers (animal-based indicators) that can be used to monitor the results of risk mitigation with biosecurity and biocontainment measures. The systematic review of risk identification and selection of biomarkers to monitor risks will lead to templates, providing a scheme from which tailor-made health&welfare plans (including biosecurity protocols) will be designed in commercial farms. The effect of the tailor-made plans on the defined indicators and their effectiveness in promoting health&welfare will be pilot-tested during 12 months on Dutch (20), Cypriotic and Greec (20) and Chinese (15) broiler farms.
This Dutch working document gives insights, derived from scientific and 'grey' literature, in risk factors for major Dutch broiler diseases (digestive, respiratory, feet disorders), in existing Dutch scoring systems, and in biomarkers (grosslist), and anticipates on the development of the risk analysis tool on health plans based on the (FAO) risk zoning as meant in T1.2 of WP1.

2. Major infectious broiler diseases in the Netherlands

[sources: Bergevoet et al, 2010; Animal Health Service https://www.gddiergezondheid.nl/pluimvee]

Table 1 *Farm related diseases in the Netherlands (including aviairy influenza because of its known risk factors and preventative measures also relevant for farm related diseases)*

Respiratory diseases*	***Digestive tract diseases****	***Foot diseases****
Infectious Coryza (acute snot)<br>[Avibacterium paragallinarium]	Necrotic enteritis<br>[Clostridium perfringens]	Enterococcus?<br>[Enterococcus cecorum]
Infectious bronchitis<br>[IB-virus]	Colibacillosis<br>[E. Coli] (secundary infection)	?
Mycoplasma gallisepticum/<br>(Mycoplasma synoviae)	Coccidiosis<br>[Eimeria maxima/tenella]	
NCD<br>[paramyxovirus](mandatory vaccination)	Gumboro disease<br>[Gumboro virus]	
Infectious Larynchotracheïtis<br>[ILT-virus]	Salmonella typhimurium/enteritidis\pullorum	
Ornythobacterium rhinotracheale (kaaskuikens)<br>[as secondary infection, less important]		
(Aviary influenza)<br>[H#N#)		

* ranked according to importance as indicated by Bergevoet et al, 2010

3. Literature scan: major diseases, transmission routes and prevention

3.1 Introduction

Search terms used on web of science are according to the major broiler diseases and/or pathogens.

3.2 Overview of diseases, risk factors and potential preventative measures

Table 2 *Risk factors and preventative measures for major broiler diseases* [only problems that affect the feet (F), respiratory system (R), digestive tract (D) of broilers are mentioned in the table; P= parasite, B=bacterium, V=virus]

Name of the disease	Type agent	Refe-rences	Risk factors	Preventative measures mentioned
**Respiratory diseases**				
Infectious bronchitis [IB-virus]	V	[1].(Ignjatovic and Sapats 2000)	Faeces, and feed and drinking water that have been contaminated by faeces are sources of infection. The virus can survive for a considerable time in faeces and is suspected to represent a continuing source of re-infection in the recovery phase of the disease. Contaminated litter,<br>footwear, clothing, utensils, equipment and personnel are all potential sources of virus for indirect transmission and have been implicated in IBV spread over large distances. The role of vertical transmission in the epidemiology of IBV has not been clearly established. Airborne (aerosols) or mechanical (via personnel, material and equipment) transmission between birds, houses and farms can take place. Movement of live birds, either as one-day-old chicks or as adult birds should be considered as a potential source for the introduction of IBV. Vectors do not appear to be a factor in the spread of IBV[1].	All-in/all-out[1]<br>Cleaning and disinfection between batches will limit the level of infection to a minimum; however, exclusion of IBV has not been achieved through such measures1<br>Vaccines[1]<br>Extreme strict SPF hygiene measures[1].
Mycoplasma infection [gallisepti-cum/ syno-viae) Different types	B	[1].(Kleven 2008).<br>[2].(Levisohn and Kleven 2000)<br>[3].(Umar, Munir et al. 2017)	Contact with contaminated dust or water particles in the air that travel via the nose or eye into the lungs. Contaminated clothes, hair, feathers, egg material (on trays) or other contaminated material. Also vertical transmission [1,3].<br>Transmission occurs vertically (in ovo), from an infected breeder flock to the progeny, or horizontally, by direct or indirect contact of susceptible birds with infected carriers or contaminated debris[2].	Prevent contact with (wild) birds[2].<br>Replacements from mycoplasma-free sources in a single-age, all-in all-out [1,3]<br>Good biosecurity[1]<br>Effective monitoring system [1,3]<br>Segregation and traffic control[3]<br>Vaccination[1]<br>Shield the stable
Infectious Laryncho-tracheitis [ILT-virus]	V	[1].(Ou and Giambrone 2012)<br>[2].(Ou, Giambrone et al. 2011)	Natural transmission of ILTV is through the upper respiratory and ocular routes and transmission between flocks occurs via contaminated equipment, humans, and litter. Sources of ILTV are clinically affected chickens, latent infected carriers, contaminated dust, litter, beetles, drinking water and fomites. Other possible sources of transmission included dog, crows, and cats [1,2].	Avoid contact between vaccinated or recovered field virus infected birds with non-vaccinated chickens[1].<br>Vaccination[1].<br>Remove contaminated fomites[1].<br>Biosecurity to prevent pathogens from infecting and transmitting disease by humans, insects, wild birds, or other animals[1].
New castle disease (NCD) [Avian Paramyxo-virus (PMV)]	V	[1].(Alexander 2000)	Contaminated water and feed, manure or secretes. Also infection via air water drops or dust. Spread from bird to bird appears to occur as the result of either inhalation of excreted droplet particles or the ingestion of infective material such as faeces. Wild birds, movements of personnel or equipment can also play a role in transmission	Vaccination [mandatory in NL][1]<br>Prevent introduction of virus[1]<br>Good hygiene (clothing change, equipment disinfection, etc.)[1]<br>Biosecurity (separate flocks, isolate hatcheries, fresh water)[1]<br>Prevent contact of material and broilers with (wild) birds[1]<br>Minimize movement on and off farm[1]<br>Disinfect all equipment (including vehicles) before entering the site[1]<br>Movements of animals and materials should take place to and from a specific collection and delivery point away from the flocks[1]
Avian Influenza [H#N#]	V	[1].(Ssematimba, Hagenaars et al. 2013)<br>2.(Thomas, Bouma et al. 2005)<br>3.(Bokma, Bergevoet et al. 2016)	Contact types are bird movements during thinning and restocking, most human movements accessing poultry houses and proximity to other poultry farms. Transmission of the virus through movements of humans (visitors, servicemen and farm personnel), vectors (wild birds, rodents, insects), air- (and dust) related routes and other fomites (e.g., delivery trucks, visitors' clothes and farm equipment) have all been hypothesized[1]. Transport of live poultry,	Depopulation of farms[2]<br>Transport ban[2]<br>Hygienic measures[2,3]<br>Reducing scavengers through covering the manure storages[1]<br>Ensuring that manure does not stay long on the premises as well as ensuring that dead birds are disposed of safely[1]

Name of the disease	Type agent	Refe-rences	Risk factors	Preventative measures mentioned
			persons and mechanical transfer of faeces of infected birds are considered to be the most important transmission routes. Consequently, these routes mainly consist of movements of people (e.g. farm owners and their staff), materials (e.g. egg trays) and vehicles (e.g. lorries that transport egg trays and eggs). Other routes of transmission are transmission from infected farms over short distances. Finally, interspecies transmission via pigs is also considered to be a source of infection to poultry[2]	Airborne contamination risks could be reduced through installation of dust extraction systems like air scrubbers[1]<br>Prevent contact with (pet) animals and insects[3]<br>Minimize visitors on farm[3]<br>Movements of animals and materials should take place to and from a specific collection and delivery point away from the flocks[3]<br>Disinfect materials and vehicles that enter the farm[3]
**Digestive tract diseases leading indirectly to foot problems**				
Coccidiosis [Eimeria maxima/ tenella]	P	[1]. (Allen and Fetterer 2002)<br>[2]. (Blake and Tomley 2014)<br>[3]. (Belli, Smith et al. 2006)<br>[4]. (Reyna, McDougald et al. 1983)	Transmission through faecal-oral route by ingestion of tissue cysts as well as oocysts that contaminate the environment[3].<br>Oocysts can also be transmitted via dust or arthropod vectors[4]	Anticoccidials[1]<br>Vaccination[1]<br>Thorough cleanout between flocks[1,2]<br>Caretakers change clothes between house[1]<br>Strict biosecurity[1]<br>Controlling house climate[2]<br>Restricting bird access to faeces[2]
Necrotic enteritis [Clostridium perfringens]	B	[1].(Cooper and Songer 2009)<br>[2].(Immerseel, Buck et al. 2004)<br>[3].(Timbermont, Lanckriet et al. 2010)<br>[4].(Moore 2016)	C. perfringens is a common intestinal inhabitant.[2]<br>Contamination of poultry feed and even vertical transmission has been suggested[1]<br>The bacterium can be found in the environment, such as soil and water. It is also shown that intestinal droppings of wild birds contain high numbers of C. perfringens[2]<br>Development of necrotic enteritis depends on the presence of predisposing factors, two of the most important being mucosal damage caused by coccidial pathogens and feed containing high protein levels[3]	Vaccination[2]<br>Coccidial vaccines and coccidiostatic drugs are able to prevent C. perfringens-associated necrotic enteritis[2]<br>The exact mechanism of infection is still unknown so the control strategy remains uncertain[4]
Colibacillosis [E.coli] secundary infection	B	[1].(Giovanardi, Campagnari et al. 2005)<br>[2].(Dho-Moulin and Fairbrother 1999)	Escherichia coli is present in the normal intestinal flora of birds. Only some strains with specific virulence attributes, designated as avian pathogenic E. coli (APEC), are able to cause disease[1].<br>Vertical transmission takes place [1,2]<br>Horizontal contamination with E. coli usually occurs through contact with other birds, or through faeces, contaminated water and feed. Birds are frequently contaminated by inhalation of particles present in dust[2]	Control of environmental contamination[2]<br>Environmental parameters such as humidity and ventilation[2]<br>Reduction of the transmission of E. coli by fumigating the eggs within 2 h after they have been laid and by discarding eggs that are cracked or those with obvious fecal contamination[2]
Salmonel-losis [Salmonella typhimurium / enteritidis/ pullorum]	B	[1]. (Van Immerseel, De Zutter et al. 2009, Totton, Farrar et al. 2012)	Contaminated dust, manure, feed, human shoes and clothing, pest animals, pet animals. Vertical transmission occurs. Introduction can take place via vehicles, people, clothing, footwear, equipment, water, feed, litter, insects, rodents, wild birds, pets, utensils and many more[1].	Introducing salmonella-free animals into your flock[1]<br>Water and feed decontamination[1]<br>Insect, rodent and vector control[1]<br>Controlled access to the farm. Only essential visitors[1]<br>Protective clothing and disinfected boots[1]<br>Simple measures such as foot baths, hand hygiene[1]<br>Minimizing movement between different animal houses[1]<br>Hygienic barriers, including anterooms[1]<br>Cleaning and disinfection after each[1]<br>Preventing contact with other animals are important[1]
Gumboro disease? [Gumboro virus]				

3.3 General literature on biosecurity of broiler farms

Belgian: Biocheck.UGent

Gelaude et al (2014) indicate that, based on scientific literature, a large number of risk factor studies related to poultry diseases is available, but always in the function of one specific disease. These authors conducted a comprehensive literature review on disease transmission in poultry in order to construct a risk-based weighting scoring system for the biosecurity level in (Belgian) broiler farms (Biocheck.Ugent). Gealude et al (2014) separated their scoring system in two main categories, external biosecurity (the introduction of off-farm pathogens) and internal biosecurity (preventing within-farm spread of pathogens), with the following (science based) content:

External biosecurity

- Location of the farm (poultry density farm vicinity)
- Purchase of 1-day-old chickens
- Removal of manure and dead animals
- Entrance of visitors and personnel
- Supply of materials (instruments, equipment)
- Supply of feed and water (and bedding materials)
- Thinning and depopulation (off-farm movement of live broilers)
- Infrastructure and biological vectors (rodents, wild birds, insects, other poultry species, other farm animal species, pets)

Internal biosecurity

- Disease management (vaccination, euthanasia policy, removing dead birds from stables, stocking density)
- Cleaning and disinfections (between flocks; before entering broiler houses)
- Materials and measures between compartments (equipments, clothing, hand washing facilities..)

In the construction of a (Dutch) risk tool concerning major respiratory, digestive tract and feet diseases in broilers, we use the Gelaude comprehensive literature review as a starting point, extend the literature review to specific major broiler diseases and identify relevant transmission routes and biosecurity measures within the separated risk zones and transition line between zones on broiler farms.

3.4 Matrix of diseases and relevant subcategories of biosecurity

In the following matrix, we have tried to relate literature insights on prevention of introduction and spread of the main Dutch broiler diseases to the subcategories of biosecurity as defined by Gelaude et al. (2014).

	**External biosecurity**								**Internal biosecurity**								
Disease	Farm location[1]	Purchase "clean" chicks	Restricted entrance[2]	Specific delivery site[3]	Supply of clean materials[4]	Prevent contact with wild birds	Prevent contact with pet/pest animals	Minimize movement on and off farm	Use of hygienic barriers[5]	Shield the stable	Use clean materials and protective clothes	Removal of manure and dead animals	All in all out system	Vaccination	Thinning and depopulation	Extreme SPF hygiene measures	Control climate in the stable
								Respiratory diseases									
**Infectious bronchitis**	x	x	x		x			x	x		x	x	x	x		x	
**Mycoplasma**	x	x	x	x	x	x		x	x	x	x	x	x	x			
**Infectious Larynchotracheïtis**	x	x	x	x	x	x	x		x		x	x	x	x			
**NCD**	x	x	x	x	x	x	x	x	x		x	x	x	x			
**Aviary influenza**	x		x	x	x	x		x	x	x	x	x			x		
								Digestive tract diseases									
**Coccidiosis**	x				x	x	x		x		x	x		x			x
**Clostridium**					x							x		x			
**Colibacillosis**		x	x		x				x		x	x					x
**Salmonella**	x	x	x	x	x	x	x	x	x	x	x	x					

[1]Farm location related to transmission by air or proximity with wild birds
[2]Restricted entrance of visitors and personnel
[3]Supply of materials from a specific delivery site on the farm
[4]Supply of clean feed, water and bedding materials
[5]Hygienic barriers, including anterooms

The overall conclusion is that many biosecurity measures are considered important at the same time for a variety of broiler diseases, both respiratory and digestive tract disorders. These various measures all help in decreasing the risk for introduction of a pathogen. To decide which measures can be applied on a

specific farm, knowledge on risk factors, disease history on farm and practical possibilities must be taken into account.

4. Existing scoring systems for biosecurity

4.1 Dutch scoring systems

IKB KIP

The IKB Kip certification scheme is a global chain quality system for the entire poultry meat sector. This allows all links involved in the production of IKB Kip poultry meat (from breeding to processing) to ensure production methods guarantees, for instance regarding the quality and safeguarding of the quality. IKB Kip is a dynamic system that is constantly developing. IKB Kip's scheme management lies with the PLUIMNED foundation (https://pluimned.avined.nl/thema/ikb-kip-in-english).

Topics in the IKB Kip certification schema for broiler farmers: Requirements on building and layout; Food safety (Salmonella check etc); Feed systems and drinking water; Animal performance and health; Veterinary medicinal products (e.g. antibiotics use and storage); Hygiene (hygiene lock, visitors protocol, cleaning and disinfection, pest control (incl. wild birds) et cetera. And special additional requirements for hygiene on broiler farms: see Appendix 1. Almost all Dutch broiler farmers participate in IKB KIP.

Dutch Hygiene Scan for poultry

(to reduce risk of introduction of avian influenza a.o., implemented in 2015; the hygiene scan is part of IKB Kip, mandatory for participating poultry farms; only Dutch version available)

Topics: Pest control and banning of wild bird; Accessibility farm yard (fences etc); Hygiene of farm site (farm yard); Farm hygiene; Barn hygiene; Hygiene regarding transport vehicles, materials and personel; Additional questions for specific farm types (e.g. hygiene requirements for farms with outdoor areas for chickens). The Hygiene scan must be scored every 12 months and be disucssed with veterinarian. Scoring system: based on expert view. Very important preventative measurements: 10 points; medium and less important measuremenst: resp. 5 and 3 points. At this moment, farmers must meet at least 60% of the total hygiene scan points to be attained.

Campylobacter scoring system

In a multi-annual study concerning reduction of Campylobacter-positive broiler flocks in the Netherlands, we developed a Campylobacter risk-analysis tool, called CAMPAS. The CAMPAS questionnaire will be further developed and validated to provide Dutch farmers with a tool to indicate the strengths and weaknesses in their biosecurity status and the associated risk for introduction of *Campylobacter* in their flocks.The questionnaire is only available in Dutch. Campylobacter remains the most common reported zoonotic pathogen in humans in the European Union since 2008 (EFSA, 2018)(EFSA, 2018)(EFSA, 2018). Poultry is a major source of human infection with Campylobacter, although the epidemiology of Campylobacter at broiler farms is still poorly understood. The purpose of the study is to investigate factors associated with the presence of Campylobacter on a selected number of broiler farms in the Netherlands.

The CAMPAS checklist follows the structure of the Dutch Hygiene scan for poultry, with additional questions related to specific risk of introduction and spreading of Campylobacter. At this moment, no

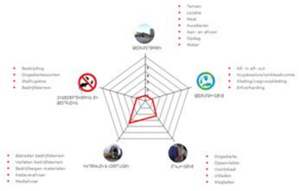

weighting factors between topics, or between questions within topics, are included. Results are presented to farmers in a 'cobweb'. The outliers are the focal point topics for strenghtening of farm biosecurity.

Scoring system footpad lesions
Footpad lesions (Michel et al. 2012); scoring system based on severity and extent of these three types of lesions:

- Type I mild lesions: scale enlargement and erythema, histologically by hyperplasia and hyperkeratosis of epidermis, superficial dermal congestion and oedema
- Type II moderate lesion: hypertrophic and hyperkeratotic scales covered with yellowish to brownish exudate, and histollogically by prominent pustular and crust-forming dermatitis
- Type III severe lesions: thick dark adherent crust, and histologically by extensive ulceration

4.2 Belgian scoring system

In our neighbour-country Belgium, the Biocheck.UGent is is an elaborate check for the biosecurity status on farm, e.g. poultry farms https://www.biocheck.ugent.be/index.php (Gelaude, Schlepers et al. 2014; see also). The university of Gent separated the Biocheck scoring system in two main categories, external biosecurity (the introduction of off-farm pathogens) and internal biosecurity (preventing within-farm spread of pathogens), of which each subcategory consist of 2 to 17 different biosecurity measures. Prioritization and weighting of the various biosecurity measures has been done by an expert panel (epidemiologists, veterinary practitioners, microbiologists, hygiene specialists) in order to indicate their relative importance. As not all transmission routes are equally efficient in disease transmission, biosecurity measures are not equally important.

The figure below illustrates the relative importance of the different pathways of pathogen transmission between farms (Laanen et al., 2010).

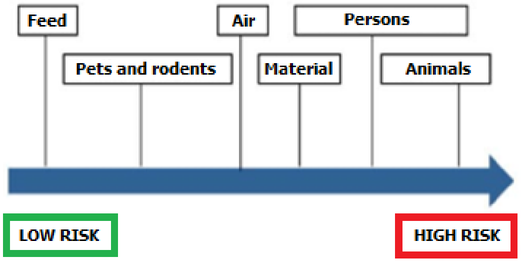

Figure 1: General arrangement of the transmission routes between farms according to their relative importance (adjusted from Boklund, 2008)

Gelaude et al (2014) emphasize that direct contact between animals (e.g. purchase of 1-day-old chicks, several age groups on the farm, free range of poultry et cetera) poses higher risks, whereas indirect contacts (e.g. rodents or equipment et cetera as vectors) are less efficient in pathogen transmission.

5. Risk zoning of broiler farms

The risk analysis tool we develop in T1.1 will be completed during the initial visit on participating broiler farms. In T1.2 we will develope health plans to reduce risks in animal friendly broiler systems. T1.2 formulates it as follows: 'Based on the results of the risk assessment **and on new insights in the design of risk zoning for broiler farms (as an elaboration of the FAO 3zone-biosecurity model)**, tailor-made health plans will be designed (proposed and discussed with each farmer-participant)'. In the development of the risk analysis tool, it is efficient to anticipate on the risk zoning in the health plans as meant in T1.2. The following picture gives an impression of the risk zoning, as worked out for a Dutch broiler farm.

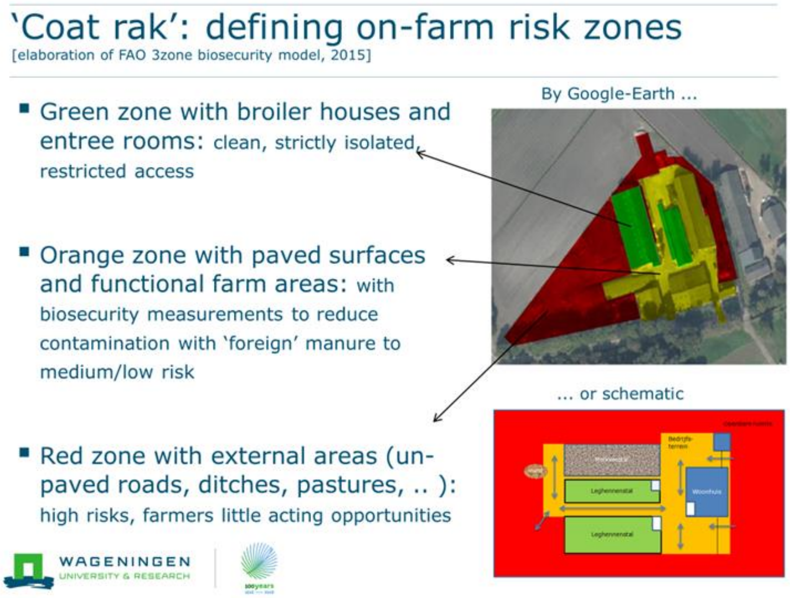

The risk analysis tool will be constructed based on the outcome of the literature scan (this document). For each zone and transition line between two zones, risk factors will be listed, objectives will be indicated and a scoring system like the Belgian Biocheck scoring system will be developed. Based on the results of the risk analysis tool, tailor-made on-farm health plans will be made to strenghten biosecurity. The following steps will be taken into account by constructing a health plan:

<u>Biosecurity</u>

- (Re)defining on-farm green-orange-red zones
- Determining hygienic measures per zone
- Determining hygienic measures when passing transition lines between zones
- Implementation of biosecurity protocols

<u>Biomarkers</u>

- Defining the biomarkers to monitor, and tailor-made objectives / atrgets for the chosen markers

6. Biomarkers

A biomarker, per definition, is a marker or indicator of a biological process or pathological states and it can provide information on a current status of future risk of disease of an individual (Pletcher et al., 2011; Moore et al., 2007). A biomarker should possess key characteristics and qualities, which will depend upon its intended use (Aronson, 2005; LaBaer, 2005). A biomarker must be accurate, sensitive and specific. The biomarker should be altered in the relevant disease and be able to discriminate between diseased and control populations. It should also be possible to quantify the biomarker reliably

and reproducibly. For diagnostic purposes biomarkers should ideally be obtained from readily accessible body fluids in animals such as blood plasma, urine, sweat and saliva or other accessible materials such as hair and feces (Moore et al., 2007)
A literature scan was performed using Web of Science and Scopus to review biomarkers that can be used in broilers to predict or indicate a disease. The following words have been used to search for literature: biomarker & chicken. This revealed a large amount of literature (respectively 217 and 343 hits, respectively). For biomarkers for oxidative stress in chicken also Google Scolar was used, because only 13 hits were found in Web of Science which were more related to stress and animal welfare. Respiratory/lung disease chicken and biomarker were used in Web of Scinece, Scopus and Google Scholar to scan literature on biomarkers for respiratory problems in chicken. The most relevant papers/reviews were selected, resulting in the following biomakers (see gross list par. 7.1). The practical feasibility is scored by two microbiologists working at Wageningen University&Research (dr. A. Rebel and dr. N. Stockhofe).

6.1 Gross list of potential biomarkers

The gross list of potential biomarkers is divided in non-specific biomarkers (6.1.1), and specific biomarkers for respectively digestive disorders (6.1.2.), respiratory disorders (6.1.3) and feet disorders (6.1.4).

6.1.1 Non-specific biomarkers

BIOMARKER	INDICATIVE OF:	MATRIX	PRACTI-CAL FEASI-BILITY (+-)
Glucocorticoïden and cathecholamines	Released by non-inflammatory and psychological stress response, by activating HPA axis and sympathic-adrenal axis. Glucocorticoïden and catecholaminses stimulate production of pro-inflammatory cytokines (IL-1, IL-6, TNFα)	Blood	
Corticotropin-releasing hormone (CRH)	Increased by activated HPA axis	Bioassay	
Cortisol/hydrocortisone [corticoïd)	Increased by activated HPA axis	Feathers	+
(Nor-)adrenaline/(nor-)epinephrine [catecholamines]	Increased by activated sympathic-adrenal axis (fight and flight)	Bioassay	-
Pro-inflammatory cytokines:	Regulating active immune responses (messengers to hepatocytes producing APP)		
Il-1β		Blood	Pcr-based
IL-6		Blood	+
TNFα	? relevant in chickens?	Blood	+
Ratio Th-1/Th-2 cytokines	Balance in pro-inflammatory/anti-inflammatory cytokines	Blood	+
Acute-phase proteins (APP):	Early detection of disturbances in homeostasis, of changes in herd health status prior to clinical disease, trauma, stress response (can be useful as infection and inflammation markers and stress indicators)	Plasma and serum	
C-reactive protein (CRP)	(first line)	Elisa	+
Serum amyloïd A protein (SAA)	(first line)	Elisa	+
Haptoglobin (Hp)	(second line)	Elisa	+
Immunoglobulins	Immune system functioning [gives little information]		
IgG	Protects against bacterial and viral infections	All body fluids	-
IgM	First antibody to fight an infection	Blood	-
Lymphocytes (white blood cell)		Blood	
Natural killer cells (NK)	Protection against virally infected cells (and tumors), activated by cytokines (interferon)	Blood	(no prac-tical tools chickens)

T-cells	Cell-mediated immunity	Blood	+
B-cells	Humoral immunity	Blood	+
Macrophages	Phagocytosis; non-specific defence (innate immunity); initiate specific defence (adapative immunity; antigen presenters tot T-cells etc.); inflammatory and anti-inflammatory (release of cytokines)	Blood	+/-
Heterophil frequencies (white blood cell counts)	The increase in heterophil frequencies measured with the hematology analyzer proved to be a very sensitive method for the measurement of changes in plasma corticosterone concentrations (in relation to stress in broiler chickens)	Blood	+
Oxidative stress	A.o indicative of activation of immune system in response to invading microorganisms (inflammation); [also following presence of xenobiotics or radiation]		
Nitric oxide (NO)	Role in activating hepatocytic cells to produce APPs. Close relationship with immune function (Chen et al. 2014)	... assay	+
Malondialdehyde (MAD)	Indicative of lipid peroxidation level (sehirli et al. 2008; Youssef et al. 2009)	Blood, tissue	+/-
Catalase (CAT)	Antioxidant enzyme that is produced naturally within the body. Activity is indicative of oxidative status (Ismail et al. 2013)		+/-
Glutathione-S-transferase (GST)	Antioxidant enzyme that is produced naturally within the body. Activity is indicative of oxidative status (Vossen et al. 2010)	Plasma	+/-
Super Oxide Dismutase (SOD)	Antioxidant enzyme that is produced naturally within the body. Activity is indicative of oxidative status (Vossen et al. 2010)	Plasma	+/-
Telomere length	Length is reducing faster during oxidative stress: telomere length might be useful as biomarker of disease progression (Houben et al. 2018)	DNA blood cells	+

6.1.2 Specific biomarkers: Digestive disorders

BIOMARKER	INDICATIVE OF:	MATRIX	PRACTICAL FEASIBILITY
Bacterial counts in liver	Increased intestinal permeability in broilers	Liver	+
LPS in serum	Lipopolysachariden, found in outer membrane of gram-negative bacteria, in serum indicative of increased intestinal paracellular permeability (IP)	Serum	+
**Fecal microbiota**: loss of species richness, and/or diversity and evenness	Poor intestinal health	Feces	-
**Enterobacteriaceae**	Dysbiosis	Blood	+
**Peptides**		Blood	
Lactoferrin, cathelicidins, defensins	Antimicrobial peptides. Indicative of damage to the intestinal epithelial barrier when secreted in increased amounts		+
Specific pathogens			
Clostridium perfringens	Necrotic enteritis	Feces	+
E. Coli	Secondary infection	Feces	+
Eimeria maxima/tenella	Coccidiosis	Feces	+
Salmonella typh./enteritidis	Salmonellosis	Feces	+
Gumboro virus	Gumboro disease	Blood	+
Campylobacter spp	Campylobacter	Feces	+
Specific antibodies			
pm			
Clinical symptoms			
Diarrhea			+

Weight changes			+
Feed-water intake/appetite			+
Mobility			+
Feathers			+
Flock activity			+
Vocalisation			+
Productivity markers			+
Feed/water intake			+
Feed conversion			+
Growth			+
Mortality			+

For potential biomarkers for assessing intestinal permeability (IP) see also Table 5 in Gilani et al., 2016:

Table 5. Summary of the different biomarkers used for assessing intestinal permeability (IP)

Test	Basic measurements	Advantage(s)	Disadvantage (s)
Lactulose (L), L-rhamnose (R) and mannitol (M) sugars	Measures transcellular (R and M) and paracellular permeability (L)	1. Measures IP throughout the intestine.<br>2. Can be used in same animals repeatedly.<br>3. Less invasive technique.	1. Has not been conducted in chickens, but has been conducted in dogs, rabbits, rats, mice, birds and humans only.<br>2. Oral gavage needs to be given with sugars to birds and requires blood collection.
Fluorescein isothiocyanate dextran (FITC-d)	Measures paracellular permeability as above (L) method	1. Has been used in chickens recently.<br>2. Measures IP throughout the intestine.<br>3. Can be used in same animals repeatedly.<br>4. Less invasive technique.	Oral gavage needs to be given with sugars to birds and require blood collection.
Ussing chamber	Trans-epithelial electrical resistance and nutrient movement	1. Many measurements can be taken from small sections of the intestines.<br>2. Can specifically measure IP of duodenum, jejunum and ileum separately.	1. *In vitro* technique as does not include the responses from live animal.<br>2. Measures IP of a specific section of the intestine at one point.<br>3. Need special apparatus.<br>4. Need to kill birds to get tissues
Tight-junction (TJ) protein expression	Measures the transcriptional process of TJ protein expressions	Has been conducted in poultry and pigs.	1. Cannot be performed in same animals repeatedly.<br>2. Not a direct measure of IP.<br>3. Messenger RNA transcription may not always lead to full protein translation.
Intestinal fatty acid binding protein (iFABP) and antitrypsin inhibitor (AAT)	Measures IP due to the damage in mucosa	1. Less invasive technique.<br>2. Can be measured in blood.<br>3. Can be used in same animals repeatedly.	Has not been conducted in chickens so no data to compare results.
D-lactate	Measures IP as lactate passes from intestine to blood	1. Less invasive technique.<br>2. Can be measured in blood.<br>3. Can be used in same animals repeatedly.	1. Only a few studies have been conducted in chickens.<br>2. Has not been used in disease- or stress-challenged chickens.
Diamine oxidase (DAO)	Measures IP as DAO passes from intestinal mucosa to blood	1. Less invasive technique.<br>2. Can be measured in blood.<br>3. Can be used in same animals repeatedly.<br>4. Has been used in intestinally compromised chickens.	Few studies have been conducted in chickens.
Bacterial translocation	Measures IP as pathogens pass from intestinal mucosa to blood	Has been used in chickens.	Need to kill birds to collect tissues.
LPS in blood	Measures IP when endotoxins of pathogens traverse from the intestinal mucosa to blood	Less invasive technique as it can be measured in blood.	1. Has been used in only one study.<br>2. The model used in the study suggests that it requires further experiments to use this model in a variety of conditions.

6.1.3 Specific biomarkers: Respiratory diseases

BIOMARKER	INDICATIVE OF:	MATRIX	PRACTICAL FEASIBILITY
Proteins			
ChPLA2_V (chicken secretory class V phospholipase A2 enzym)	Lung inflammation (novel broad biomarker of infectious bronchitis) [Karray et al, 2012] was found to display potent Gram-positive and Gram-negative bactericidal activity and antifungal activity *in vitro*	Lung, spleen	

Specific pathogens			
Salmonella enteritidis			
pm			
Specific antibodies		Elisa	+
Pm			
Clinical symptoms			
Tracheal rales, coughing, sneezing			
Productivity markers			
Feed/water intake			+
Feed conversion			+
Growth			+
Mortality			+

6.1.4 Specific biomarkers: Feet disorders

BIOMARKER	INDICATIVE OF:	MATRIX	PRACTICAL FEASIBILITY
Visual scoring of footpad lesions	Footpad lesions (Michel et al. 2012)		
Type I mild lesions: scale enlargement and erythema, histologically by hyperplasia and hyperkeratosis of epidermis, superficial dermal congestion and oedema	[scoring system based on severity and extent of these three types of lesions]	Visual	+
Type II moderate lesion: hypertrophic and hyperkeratotic scales covered with yellowish to brownish exudate, and histollogically by prominent pustular and crust-forming dermatitis		Visual	+
Type III severe lesions: thick dark adherent crust, and histologically by extensive ulceration		Visual	+
Litter humidity	Predictive of footpad dermatitis		+
pm			
Specific pathogens			-
Pm			
Specific antibodies		Elisa	+
pm			
Productivity markers			
Feed/water intake			+
Feed conversion			+
Movement/mobility			+

7. Follow-up

The result of the literature scan on risk factors, scoring systems and biomarkers forms the basis for the construction of a risk tool and for selecting biomarkers to be used during the field trial with broiler farms.

Literature

Literature scan risk factors and preventative measures

Alexander, D. (2000). "Newcastle disease and other avian paramyxoviruses." Revue Scientifique et Technique-Office International des Epizooties **19**(2): 443-455.

Allen, P. C. and R. Fetterer (2002). "Recent advances in biology and immunobiology of Eimeria species and in diagnosis and control of infection with these coccidian parasites of poultry." Clinical microbiology reviews **15**(1): 58-65.

Belli, S. I., et al. (2006). "The coccidian oocyst: a tough nut to crack!" Trends in parasitology **22**(9): 416-423.

Bergevoet, R., et al. (2010). Bedrijfsgebonden dierziekten op varkens-, rundvee-en pluimveebedrijven, Wageningen UR Livestock Research.

Blake, D. P. and F. M. Tomley (2014). "Securing poultry production from the ever-present Eimeria challenge." Trends in parasitology **30**(1): 12-19.

Bokma, M., et al. (2016). Handelingsperspectief voor pluimveehouders in de preventie van laag-en hoogpathogene vogelgriep (AI), Wageningen Livestock Research.

Cooper, K. K. and J. G. Songer (2009). "Necrotic enteritis in chickens: a paradigm of enteric infection by Clostridium perfringens type A." Anaerobe **15**(1-2): 55-60.

Dho-Moulin, M. and J. M. Fairbrother (1999). "Avian pathogenic Escherichia coli (APEC)." Veterinary research **30**(2-3): 299-316.

Gelaude, P., et al. (2014). "Biocheck. UGent: A quantitative tool to measure biosecurity at broiler farms and the relationship with technical performances and antimicrobial use." Poultry science **93**(11): 2740-2751.

Giovanardi, D., et al. (2005). "Avian pathogenic Escherichia coli transmission from broiler breeders to their progeny in an integrated poultry production chain." Avian Pathology **34**(4): 313-318.

Ignjatovic, J. and S. Sapats (2000). "Avian infectious bronchitis virus." Revue Scientifique et Technique-Office International des Epizooties **19**(2): 493-501.

Immerseel, F. V., et al. (2004). "Clostridium perfringens in poultry: an emerging threat for animal and public health." Avian Pathology **33**(6): 537-549.

Kleven, S. (2008). "Control of avian mycoplasma infections in commercial poultry." Avian diseases **52**(3): 367-374.

Levisohn, S. and S. Kleven (2000). "Avian mycoplasmosis (Mycoplasma gallisepticum)." Revue Scientifique et Technique-Office International des Epizooties **19**(2): 425-434.

Moore, R. J. (2016). "Necrotic enteritis predisposing factors in broiler chickens." Avian Pathology **45**(3): 275-281.

Ou, S.-C. and J. J. Giambrone (2012). "Infectious laryngotracheitis virus in chickens." World journal of virology **1**(5): 142.

Ou, S., et al. (2011). "Infectious laryngotracheitis vaccine virus detection in water lines and effectiveness of sanitizers for inactivating the virus." Journal of Applied Poultry Research **20**(2): 223-230.

Reyna, P. S., et al. (1983). "Survival of coccidia in poultry litter and reservoirs of infection." Avian diseases: 464-473.

Ssematimba, A., et al. (2013). "Avian influenza transmission risks: analysis of biosecurity measures and contact structure in Dutch poultry farming." Preventive veterinary medicine **109**(1-2): 106-115.

Thomas, M., et al. (2005). "Risk factors for the introduction of high pathogenicity Avian Influenza virus into poultry farms during the epidemic in the Netherlands in 2003." Preventive veterinary medicine **69**(1-2): 1-11.

Timbermont, L., et al. (2010). "Control of Clostridium perfringens-induced necrotic enteritis in broilers by target-released butyric acid, fatty acids and essential oils." Avian Pathology **39**(2): 117-121.

Totton, S. C., et al. (2012). "A systematic review and meta-analysis of the effectiveness of biosecurity and vaccination in reducing Salmonella spp. in broiler chickens." Food research international **45**(2): 617-627.

Umar, S., et al. (2017). "Mycoplasmosis in poultry: update on diagnosis and preventive measures." World's Poultry Science Journal **73**(1): 17-28.

Van Immerseel, F., et al. (2009). "Strategies to control Salmonella in the broiler production chain." World's Poultry Science Journal **65**(3): 367-392.

Literature scan biomarkers

Ahmad, M. Z., A. Khan, M. T. Javed and I. Hussain (2015). "Impact of chlorpyrifos on health biomarkers of broiler chicks." Pesticide Biochemistry and Physiology **122**: 50-58.

Amid, A., N. A. Samah and F. Yusof (2012). "Identification of troponin I and actin, alpha cardiac muscle 1 as potential biomarkers for hearts of electrically stimulated chickens." Proteome Science **10**(1).

Armorini, S., K. M. Al-Qudah, A. Altafini, A. Zaghini and P. Roncada (2015). "Biliary ochratoxin A as a biomarker of ochratoxin exposure in laying hens: An experimental study after administration of contaminated diets." Research in Veterinary Science **100**: 265-270.

Ayo, J. O., H. K. Makeri, N. S. Minka and T. Aluwong (2018). "Circadian rhythms of biomarkers of oxidative stress and their characteristics in broiler chickens reared under natural light/dark cycle." Biological Rhythm Research **49**(1): 119-127.

Bargar, T. A., G. I. Scott and G. P. Cobb (2003). "Chorioallantoic membranes indicate avian exposure and biomarker responses to environmental contaminants: A laboratory study with white leghorn chickens (Gallus domesticus)." Environmental Science & Technology **37**(2): 256-260.

Bateson, M. (2016). "Cumulative stress in research animals: Telomere attrition as a biomarker in a welfare context?" BioEssays **38**(2): 201-212.

Beauclercq, S., L. Nadal-Desbarats, C. Hennequet-Antier, I. Gabriel, S. Tesseraud, F. Calenge, E. Le Bihan-Duval and S. Mignon-Grasteau (2018). "Relationships between digestive efficiency and metabolomic profiles of serum and intestinal contents in chickens." Scientific Reports **8**(1).

Bedanova, I., E. Voslarova, G. Zelinska, J. Blahova, P. Marsalek and J. Chloupek (2014). "Neopterin and biopterin as biomarkers of immune system activity associated with crating in broiler chickens." Poultry Science **93**(10): 2432-2438.

Belardi, J. A. and M. Albertal (2015). "Elevated biomarkers and contrast-induced acute kidney failure: What comes first the chicken or the egg?" Catheterization and Cardiovascular Interventions **85**(3): 343-344.

Boulton, K., Z. Wu, A. Psifidi and D. Hume (2016). "The potential of serum IL-10 as a diagnostic biomarker of resilience in the domestic chicken to infection from Eimeria Spp." Journal of Animal Science **94**: 158-159.

Cahyaningsih, U., A. S. Satyaningtijas, R. Tarigan and A. B. Nugraha (2018). Chicken I-FABP as biomarker of chicken intestinal lesion caused by coccidiosis. IOP Conference Series: Earth and Environmental Science.

Chen, J., K. Chen, S. Yuan, X. Peng, J. Fang, F. Wang, H. Cui, Z. Chen, J. Yuan and Y. Geng (2016). "Effects of aflatoxin B<inf>1</inf>on oxidative stress markers and apoptosis of spleens in broilers." Toxicology and Industrial Health **32**(2): 278-284.

Chen, J., G. Tellez and J. Escobar (2016). "Identification of Biomarkers for Footpad Dermatitis Development and Wound Healing." Frontiers in Cellular and Infection Microbiology **6**.

Chen, Z. S., A. Krieger, Y. Liu, J. Ross and R. Krieger (2015). "Fecal DDA as a biomarker of DDT exposure in chickens." Toxicological and Environmental Chemistry **97**(7): 946-960.

Chen, Z. S., O. Unoje, L. Cui, K. Aratani and R. I. Krieger (2009). "DDA in chickens, a pilot study as a DDT biomarker." Abstracts of Papers of the American Chemical Society **238**: 422-422.

Chowdhury, V. S. (2019). "Heat Stress Biomarker Amino Acids and Neuropeptide Afford Thermotolerance in Chicks." Journal of Poultry Science **56**(1): 1-11.

Cook, N. J., R. Renema, C. Wilkinson and A. L. Schaefer (2009). "Comparisons among serum, egg albumin and yolk concentrations of corticosterone as biomarkers of basal and stimulated adrenocortical activity of laying hens." British Poultry Science **50**(5): 620-633.

Corzo, A., M. T. Kidd, G. T. Pharr and S. C. Burgess (2004). "Initial mapping of the chicken blood plasma proteome." International Journal of Poultry Science **3**(3): 157-162.

Dong, J. Q., H. Zhang, X. F. Jiang, S. Z. Wang, Z. Q. Du, Z. P. Wang, L. Leng, Z. P. Cao, Y. M. Li, P. Luan and H. Li (2015). "Comparison of serum biochemical parameters between two broiler chicken lines divergently selected for abdominal fat content." Journal of Animal Science **93**(7): 3278-3286.

Dong, J. Q., X. Y. Zhang, S. Z. Wang, X. F. Jiang, K. Zhang, G. W. Ma, M. Q. Wu, H. Li and H. Zhang (2018). "Construction of multiple linear regression models using blood biomarkers for selecting against abdominal fat traits in broilers." Poultry Science **97**(1): 17-23.

Fletcher, O. J., X. Tan, L. Cortes and I. Gimeno (2012). "Cost effective and time efficient measurement of CD4, CD8, major histocompatibility complex Class II, and macrophage antigen expression in the lungs of chickens." Veterinary Immunology and Immunopathology **146**(3-4): 225-236.

Garner, C., S. Smith, N. C. Elviss, T. J. Humphrey, P. White, N. M. Ratcliffe and C. S. Probert (2008). "Identification of Campylobacter infection in chickens from volatile faecal emissions." Biomarkers **13**(4): 413-421.

Ghareeb, K., K. Konig, W. A. Awad, Q. Zebeli and J. Bohm (2015). "The impact of a microbial feed supplement on small intestine integrity and oxidative stress biomarker in broiler chickens." Avian Biology Research **8**(3): 185-189.

Gilani, S., G. S. Howarth, S. M. Kitessa, R. E. A. Forder, C. D. Tran and R. J. Hughes (2016). "New biomarkers for intestinal permeability induced by lipopolysaccharide in chickens." Animal Production Science **56**(12): 1984-1997.

Gilani, S., G. S. Howarth, S. M. Kitessa, C. D. Tran, R. E. A. Forder and R. J. Hughes (2017). "New biomarkers for increased intestinal permeability induced by dextran sodium sulphate and fasting in chickens." Journal of Animal Physiology and Animal Nutrition **101**(5): e237-e245.

Hajimohammadi, A., H. Rajaian, E. Khaliji, S. Nazifi and M. Ansari-Lari (2014). "Serum cardiac troponin I as a biomarker in cardiac degeneration following experimental salinomycin toxicosis in sheep." Veterinarski Arhiv **84**(1): 41-51.

He, H. Q., K. J. Genovese, C. L. Swaggerty, D. J. Nisbet and M. H. Kogut (2013). "Nitric Oxide as a Biomarker of Intracellular Salmonella Viability and Identification of the Bacteriostatic Activity of Protein Kinase A Inhibitor H-89." Plos One **8**(3).

Ishii, C., Y. Ikenaka, O. Ichii, S. M. M. Nakayama, S. I. Nishimura, T. Ohashi, M. Tanaka, H. Mizukawa and M. Ishizuka (2018). "A glycomics approach to discover novel renal biomarkers in birds by administration of cisplatin and diclofenac to chickens." Poultry Science **97**(5): 1722-1729.

Ismail, I. B., K. A. Al-Busadah and S. M. El-Bahr (2013). "Oxidative stress biomarkers and biochemical profile in broilers chicken fed zinc bacitracin and ascorbic acid under hot climate." American Journal of Biochemistry and Molecular Biology **3**(2): 202-214.

Kamboh, A. A., S. Q. Hang, M. Bakhetgul and W. Y. Zhu (2013). "Effects of genistein and hesperidin on biomarkers of heat stress in broilers under persistent summer stress." Poultry Science **92**(9): 2411-2418.

Karray, A., Y. Ben Ali, J. Boujelben, S. Amara, F. Carrire, Y. Gargouri and S. Bezzine (2012). "Drastic changes in the tissue-specific expression of secreted phospholipases A2 in chicken pulmonary disease." Biochimie **94**(2): 451-460.

Li, J. L. and R. A. Sunde (2016). "Selenoprotein transcript level and enzyme activity as biomarkers for selenium status and selenium requirements of chickens (Gallus gallus)." PLoS ONE **11**(4).

Mountzouris, K. C., C. Balaskas, I. Xanthakos, A. Tzivinikou and K. Fegeros (2009). "Effects of a multi-species probiotic on biomarkers of competitive exclusion efficacy in broilers challenged with Salmonella enteritidis." British Poultry Science **50**(4): 467-478.

Mountzouris, K. C., P. Tsitrsikos, I. Palamidi, A. Arvaniti, M. Mohnl, G. Schatzmayr and K. Fegeros (2010). "Effects of probiotic inclusion levels in broiler nutrition on growth performance, nutrient digestibility, plasma immunoglobulins, and cecal microflora composition." Poultry Science **89**(1): 58-67.

Niewold, T. A. (2015). Intestinal health biomarkers in vivo. Intestinal Health: Key to Maximise Growth Performance in Livestock**:** 219-228.

Oskoueian, E., P. D. Eckersall, E. Bencurova and T. Dandekar (2016). Application of proteomic biomarkers in livestock disease management. Agricultural Proteomics Volume 2: Environmental Stresses**:** 299-310.

Palamidi, I., K. Fegeros, M. Mohnl, W. H. A. Abdelrahman, G. Schatzmayr, G. Theodoropoulos and K. C. Mountzouris (2016). "Probiotic form effects on growth performance, digestive function, and immune related biomarkers in broilers." Poultry Science **95**(7): 1598-1608.

Paraskeuas, V., K. Fegeros, I. Palamidi, C. Hunger and K. C. Mountzouris (2017). "Growth performance, nutrient digestibility, antioxidant capacity, blood biochemical biomarkers and cytokines expression in broiler chickens fed different phytogenic levels." Animal Nutrition **3**(2): 114-120.

Park, B. S., Y. K. Oh, M. J. Kim and W. B. Shim (2014). "Skeletal Muscle Troponin I (TnI) in Animal Fat Tissues to Be Used as Biomarker for the Identification of Fat Adulteration." Korean Journal for Food Science of Animal Resources **34**(6): 822-828.

Rath, N. C., N. B. Anthony, L. Kannan, W. E. Huff, G. R. Huff, H. D. Chapman, G. F. Erf and P. Wakenell (2009). "Serum ovotransferrin as a biomarker of inflammatory diseases in chickens." Poultry Science **88**(10): 2069-2074.

Roque, K., K. M. Shin, J. H. Jo, H. A. Kim and Y. Heo (2015). "Relationship between chicken cellular immunity and endotoxin levels in dust from chicken housing environments." Journal of Veterinary Science **16**(2): 173-177.

Shah, A. K., K. A. Lêao, E. Choi, D. Chen, B. Gautier, D. Nancarrow, D. C. Whiteman, N. A. Saunders, A. P. Barbour, V. Joshi and M. M. Hill (2015). "Serum glycoprotein biomarker discovery and qualification pipeline reveals novel diagnostic biomarker candidates for esophageal adenocarcinoma." Molecular and Cellular Proteomics **14**(11): 3023-3039.

So, H. K., P. K. Mandal, M. O. Baatartsogt, H. K. Lim, C. H. Lee, J. H. Lee and K. Choi (2009). "Biomarkers identified by proteomic study of spleen lymphocyte from broilers infected with Salmonella gallinarum after feeding Korean mistletoe (Viscurn album coloratum)." Asian Journal of Animal and Veterinary Advances **4**(3): 148-159.

Soares, B. R., A. P. A. Souza, D. B. Prates, C. I. de Oliveira, M. Barral-Netto, J. C. Miranda and A. Barral (2013). "Seroconversion of sentinel chickens as a biomarker for monitoring exposure to visceral Leishmaniasis." Scientific Reports **3**.

Tsai, M. T., Y. J. Chen, C. Y. Chen, M. H. Tsai, C. L. Han, Y. J. Chen, H. J. Mersmann and S. T. Ding (2017). "Identification of potential plasma biomarkers for nonalcoholic fatty liver disease by integrating transcriptomics and proteomics in laying HENS." Journal of Nutrition **147**(3): 293-303.

Tyagi, P., D. R. Edwards and M. S. Coyne (2009). "Fecal sterol and bile acid biomarkers: Runoff concentrations in animal waste-amended pastures." Water, Air, and Soil Pollution **198**(1-4): 45-54.

Wang, W., M. Chen, X. Jin, X. Li, Z. Yang, H. Lin and S. Xu (2018). "H2S induces Th1/Th2 imbalance with triggered NF-κB pathway to exacerbate LPS-induce chicken pneumonia response." Chemosphere **208**: 241-246.

Xu, H., Y. Yao, Y. Zhao, L. P. Smith, S. J. Baigent and V. Nair (2008). "Analysis of the expression profiles of Marek's disease virus-encoded microRNAs by real-time quantitative PCR." Journal of Virological Methods **149**(2): 201-208.

Xu, L., Y. He, Y. Ding, G. E. Liu, H. Zhang, H. H. Cheng, R. L. Taylor and J. Song (2018). "Genetic assessment of inbred chicken lines indicates genomic signatures of resistance to Marek's disease." Journal of Animal Science and Biotechnology **9**(1).

You, X., M. Xu, Q. Li, K. Zhang, G. Hao and H. Xu (2019). "Discovery of potential transcriptional biomarkers in broiler chicken for detection of amantadine abuse based on RNA sequencing technology." Food Additives and Contaminants - Part A Chemistry, Analysis, Control, Exposure and Risk Assessment.

Zhang, H. L., Z. Q. Xu, L. L. Yang, Y. X. Wang, Y. M. Li, J. Q. Dong, X. Y. Zhang, X. Y. Jiang, X. F. Jiang, H. Li, D. X. Zhang and H. Zhang (2018). "Genetic parameters for the prediction of abdominal fat traits using blood biochemical indicators in broilers." British Poultry Science **59**(1): 28-33.

Zhang, Y. H., Z. Liu, R. R. Liu, J. Wang, M. Q. Zheng, Q. H. Li, H. X. Cui, G. P. Zhao and J. Wen (2018). "Alteration of hepatic gene expression along with the inherited phenotype of acquired fatty liver in chicken." Genes **9**(4).

Zhong, X., S. Gao, J. J. Wang, L. Dong, J. Huang, L. L. Zhang and T. Wang (2014). "Effects of linseed oil and palm oil on growth performance, tibia fatty acid and biomarkers of bone metabolism in broilers." British Poultry Science **55**(3): 335-342.

Appendix 1 Hygiene regulations in Ducth broiler qualisystem (IKB KIP)

**FEED (SYSTEMS) AND DRINKING WATER (SYSTEMS)**	
Broilers have acces to feed and water without limitation.	
**ANIMAL PERFORMANCE AND HEALTH**	
The poultry farmer must ensure that the vet(erinary practice) visits the business at least once in each cycle.	For a clinical inspection and operational guidance, including an evaluation of the antibiotics use, if any. Demonstrate using the vet's report.
All chickens present in one stable are supplied at the same time.	Exception is the placement of by-products (originating from greatgrandparent animal companies).
**HYGIENE**	
All visitors who enter the clean area of the company property must use the hygiene lock before entering.	A transitional period is in force: 1-3-2020. Only persons who ride the transport vehicles and who enter the clean area of company building may drive over the company premises without using the hygiene lock first.
The hygiene lock is located outside the stable (animal area), preferably next to the entrance to the company premises. An entree lock is equipped with a separate entrance and exit and a physical barrier between the clean and unclean parts of the hygiene lock.	A transitional period is in force: 1-3-2020. The physical barrier is preferably a door, but can also be a bench, for example. A plank is not permitted
The hygiene lock is equipped with a shower, an area where people can change into clean clothing/footwear and a hand-wash facility with a sink with drainage, water, soap and a (disposable) towel.	A transitional period is in force: 1-3-2020. Next to clean clothing and footware, there are additional requirements if there is no working shower available. Vistitors are required to wear a hairnet and mouth mask. IKB PSB companies may use their own clothing and footwear as long as these are clean. The hand-wash facility is preferably located in the clean area of the hygiene lock and as close as possible to the physical barrier. Hands must preferably be washed before one changes into clean company clothing/footwear.
After the operator has cleaned and disinfected the barn and before a new flock is introduced, he/she must have a hygienogram carried out once per calendar year.	Sampling and analysis is performed in accordance with the HOSOSO-regulation AVINED.
If the result of the hygienogram in VG04 is > 1.5, the stable must be sufficiently cleaned and disinfected before installation of no later than the second new pair, so that the result of the hygienogram is < 1.5. Only if the result of the hygienogram is < 1.5 in one of the two vacancies, then may the stable pair be placed in the stable.	Given the often tight planning, the broiler farmer is given two chances to obtain a hygienogram with a result of < 1.5. If this has not been obtained, then the installation of the second new pair must be postponed until the result is < 1.5.
