# Supplementary figures and images for: Development and implementation of a risk assessment tool for broiler farm biosecurity and a health intervention plan in the Netherlands, Greece, and Cyprus
Source: Poult Sci. 2022 Dec 9;102(2):102394. doi: 10.1016/j.psj.2022.102394 (PMC9811215; doi:10.1016/j.psj.2022.102394)

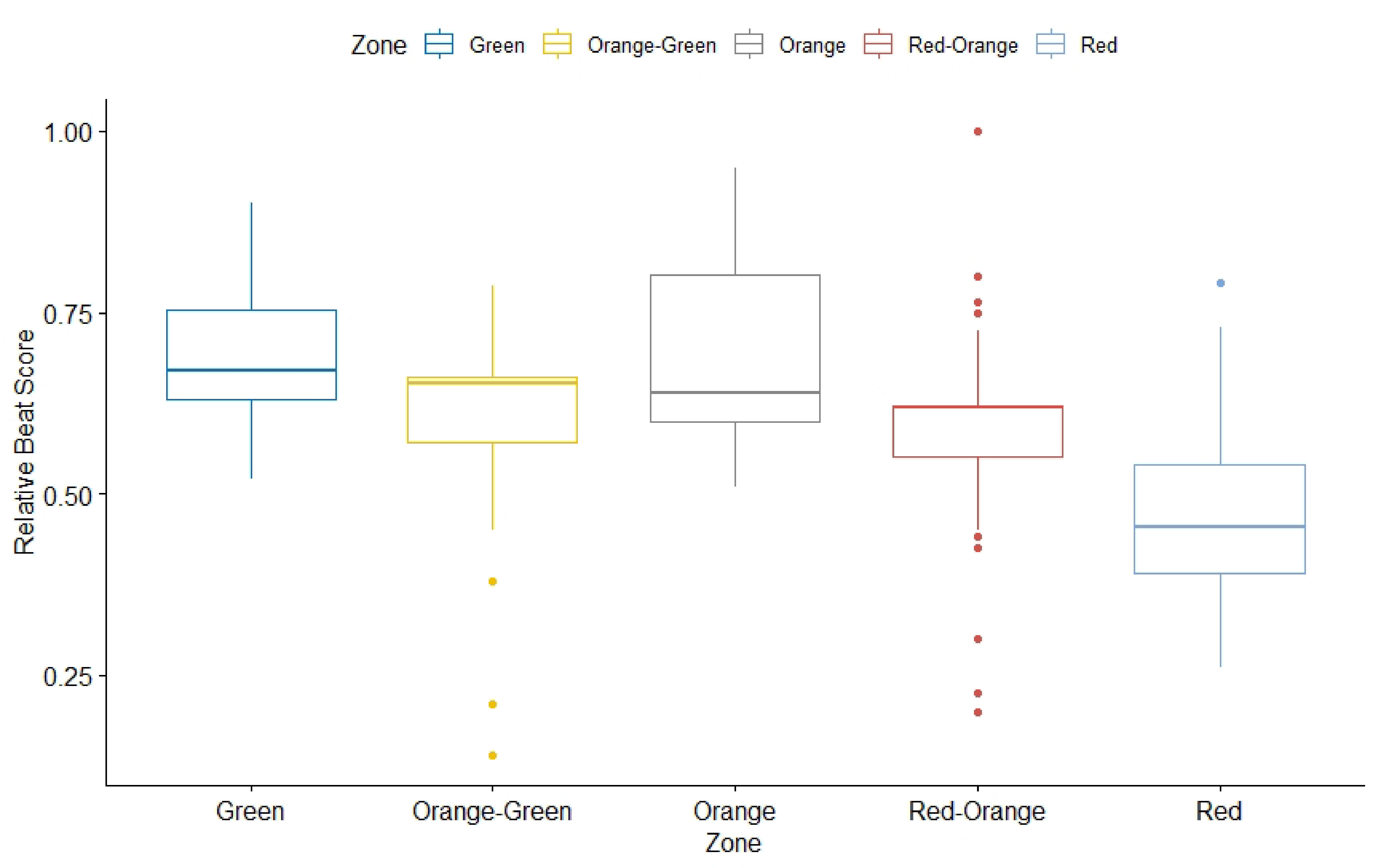

Supplement: Supplementary file 4 [file mmc4.jpg]
